# *Chrysosplenium ningqiangense* (Saxifragaceae), a new species from Shaanxi Province, China

**DOI:** 10.3897/phytokeys.272.184683

**Published:** 2026-04-01

**Authors:** Cai-Quan Shen, Yong Wang, Ke-Hui Zhang, Tian-Ge Yang, Rui Qin, Hong Liu

**Affiliations:** 1 Hubei Provincial Key Laboratory for Protection and Application of Special Plant Germplasm in Wuling Area of China, Key Laboratory of State Ethnic Affairs Commission for Biological Technology, College of Life Sciences, South-Central Minzu University, Wuhan 430074, Hubei province, China South-Central Minzu University Wuhan China; 2 School of Biological Science and Engineering, Shaanxi University of Technology, Hanzhong 723001, Shaanxi Province, China Shaanxi University of Technology Hanzhong China

**Keywords:** *
Chrysosplenium
*, morphology, new species, phylogeny, taxonomy

## Abstract

*Chrysosplenium ningqiangense* (Saxifragaceae) is described as a new species discovered in Hanshuiyuan Forest Park, Ningqiang County, Shaanxi Province, China. Morphologically, it resembles *C. serreanum*, *C. alternifolium* and *C. japonicum*, but can be distinguished by its leaf shape, yellow and loose inflorescences, absence of stolons, bulbils or bulbs at the stem base, and reddish-brown seeds with a tessellate and papillate surface. Phylogenetic analyses, based on ITS and cpDNA sequences, indicate that *C. ningqiangense* clusters with *C. hydrocotylifolium*, *C. guangxiense*, *C. macrophyllum* and *C. zhangjiajieense*. Although these species differ in morphological characteristics, phylogenetic evidence confirms *C. ningqiangense* as a distinct species within the subgenus *Gamosplenium*.

## Introduction

*Chrysosplenium* L. (1753) is a genus of small perennial herbs, characterised by flagellate stolons or bulbs, belonging to the Saxifragaceae family (Bensel and Palser 1975; Pan and Ohba 2001; Kim et al. 2018, 2019). The genus comprises approximately 72 species, primarily distributed in Asia, Europe, and North America, with a few species found in temperate regions of the Southern Hemisphere (Hara 1957; Kim et al. 2019; Wu et al. 2020; Yang et al. 2023; Jang et al. 2024; Xiang et al. 2024). In China, about 40 species have been recorded, representing over 56% of the genus, with 25 species endemic to the country (Pan and Ohba 2001; Liu et al. 2016; Kim et al. 2019; Fu et al. 2020, 2021, 2024).

Morphologically, *Chrysosplenium* is distinguished by its tetramerous flowers, petaloid sepals, and four or eight stamens in the Saxifragaceae family (Maximowicz 1872; Kim et al. 2018; Jang et al. 2024). However, species delimitation within the genus remains challenging due to morphological variations influenced by growth periods and habitats (Kim et al. 2019; Jang et al. 2024). Hara (1957) classified the genus into two groups and 17 series based on the morphological characteristics, while Pan and Ohba (2001) divided the Chinese *Chrysosplenium* into two subgenera, including five sections and 10 series. Phylogenetic studies have considerably confirmed the classification proposed by Hara (Nakazawa et al. 1997; Soltis et al. 2001). However, there is a continuous debate regarding the origin of the genus. Hara (1957) suggested that *Chrysosplenium* originated in South America with subsequent migration to North America and the Old World. In contrast, Soltis et al. (2001) speculated that the genus may have originated in East Asia, with several independent migration events to the New World.

The Hanshuiyuan Forest Park in Ningqiang County, Shaanxi Province, is the origin of the Han River in China. During a plant diversity survey in the Han River basin in July 2020, Dr. Wang from Shaanxi University of Technology discovered a new *Chrysosplenium* species in Hanshuiyuan Forest Park and provided us with photographs for identification. Initial observations suggested morphological similarities to *C. alternifolium* L., *C. serreanum* Hand.-Mazz. and *C. japonicum* (Maxim.) Makino, but distinct differences in leaf shape and stolon structure were noted. After two years of observation, including flowering and fruiting stages and microscopic photography of seeds, the species was confirmed as a new species and named *Chrysosplenium ningqiangense*. Phylogenetic analyses further supported its classification as a new species within Subgen. *Gamosplenium*. The discovery of this new species not only enriches the species diversity of *Chrysosplenium*, but also provides new insights into the phylogenetic relationships amongst species within the genus.

## Materials and methods

### Morphological observations and conservation assessments

The morphological characterisation of the new species was based on living plants and herbarium specimens. Seed morphology was examined using scanning electron microscopy (SEM). Seeds were collected from the field and dried using silica gel. Pre-treatment procedures, including impurity removal, air-drying and gold-coating, were performed according to the methods described by Fu et al. (2020). Observations and photographs were taken using a Hitachi SU8010 scanning electron microscope. At least 15 seeds were examined to ascertain their size and ornamentation. A conservation assessment was conducted following the guidelines of the IUCN (2022).

### Phylogenetic reconstruction

We downloaded all published nrITS sequences and chloroplast genomes of *Chrysosplenium* from NCBI. A total of 48 chloroplast genomes and 54 nrITS sequences (including *C. ningqiangense*) were obtained for phylogenetic analysis to determine the phylogenetic position of the new species within *Chrysosplenium* (Table 1). *Peltoboykinia tellimoides* (Maxim.) H. Hara was used as an outgroup, following Jang et al. (2024) and Fu et al. (2024).

**Table 1.** GenBank accession numbers of taxa included in phylogenetic analysis. Sequences generated in this study are marked with asterisks (*). Missing data are indicated with “–”.

Species	ITS	cpDNA
*Chrysosplenium album*	OP154009.1	NC067019.1
*Chrysosplenium alpinum*	OP154056.1	NC084130.1
*Chrysosplenium alternifolium*	OP154010.1	NC051986.1
*Chrysosplenium aureobracteatum*	MK989508.1	NC039740.1
*Chrysosplenium axillare*	OP154014.1	NC067016.1
*Chrysosplenium biondianum*	OP154015.1	NC067007.1
*Chrysosplenium carnosum*	OP154016.1	NC067026.1
*Chrysosplenium chinense*	MK402029.1	–
*Chrysosplenium davidianum*	OP154017.1	NC067002.1
*Chrysosplenium delavayi*	OP154018.1	NC067004.1
*Chrysosplenium dubium*	OP154019.1	–
*Chrysosplenium echinus*	OP154020.1	NC067020.1
*Chrysosplenium fallax*	–	NC084262.1
*Chrysosplenium fauriei*	OP154021.1	NC067023.1
*Chrysosplenium flagelliferum*	OP154023.1	NC067006.1
*Chrysosplenium flaviflorum*	MK989515.1	–
*Chrysosplenium forrestii*	OP154024.1	NC067027.1
*Chrysosplenium giraldianum*	OP154025.1	NC067011.1
*Chrysosplenium glossophyllum*	OP154026.1	NC067008.1
*Chrysosplenium grayanum*	OP154027.1	NC067018.1
*Chrysosplenium griffithii*	OP154028.1	NC067010.1
Chrysosplenium griffithii var. intermedium	OP154029.1	OK336543.1
*Chrysosplenium guangxiense*	OR941245.1	OP093635.1
*Chrysosplenium henryi*	OP154030.1	NC066997.1
*Chrysosplenium hydrocotylifolium*	OP154031.1	NC067005.1
*Chrysosplenium japonicum*	OP154032.1	NC067017.1
*Chrysosplenium jienningense*	MK402032.1	–
*Chrysosplenium kamtschaticum*	OP154033.1	NC051988.1
*Chrysosplenium kiotense*	OP154034.1	NC067021.1
*Chrysosplenium lanuginosum*	OP154035.1	NC066999.1
*Chrysosplenium lectus-cochleae*	OP154036.1	NC067013.1
*Chrysosplenium macrophyllum*	OP154037.1	MK973001.3
*Chrysosplenium macrospermum*	OP154038.1	NC067024.1
*Chrysosplenium macrostemon*	OP154039.1	NC067022.1
*Chrysosplenium microspermum*	OP154042.1	NC067009.1
*Chrysosplenium nepalense*	OP154043.1	NC067000.1
Chrysosplenium ningqiangense*	PQ777469.1	PQ783041.1
*Chrysosplenium nudicaule*	OP154044.1	MZ424445.1
*Chrysosplenium oppositifolium*	OP154057.1	NC084131.1
*Chrysosplenium pilosum*	OP154046.1	NC067001.1
Chrysosplenium pilosum var. fulvum	MK989507.1	–
Chrysosplenium pilosum var. sphaerospermum	JN375562.1	–
*Chrysosplenium qinlingense*	OP154047.1	NC067012.1
*Chrysosplenium ramosum*	OP154048.1	MK973002.2
*Chrysosplenium sedakowii*	OP154049.1	–
*Chrysosplenium serreanum*	OP154050.1	NC067003.1
*Chrysosplenium sinicum*	OP154051.1	NC051987.1
*Chrysosplenium taibaishanense*	OP154052.1	NC067015.1
*Chrysosplenium tetrandrum*	OP154058.1	NC084132.1
*Chrysosplenium uniflorum*	OP154053.1	NC066998.1
*Chrysosplenium valdepilosum*	MK989512.1	–
*Chrysosplenium valdivicum*	OP154060.1	NC084134.1
*Chrysosplenium wrightii*	OP154059.1	NC084133.1
*Chrysosplenium zhangjiajieense*	OP154054.1	NC067025.1
*Chrysosplenium zhouzhiense*	OP154055.1	NC067014.1
*Peltoboykinia tellimoides*	JQ895246.1	MZ779205.1

DNA extraction for *C. ningqiangense* was performed from silica-gel-dried leaf fragments using the modified 2×CTAB procedure of Doyle and Doyle (1987). All sequences were obtained from genome skimming data. DNA extraction, library preparation, and sequencing were conducted at BENAGEN Company (Wuhan, China). The nrDNA regions (18S-ITS1-5.8S-ITS2-26S) and the complete chloroplast genome were assembled using GetOrganelle v1.7.4 with default parameters (Jin et al. 2020). Annotation was carried out using Geseq (Tillich et al. 2017) and Geneious 11.0.4 (Kearse et al. 2012). The final nrITS and chloroplast genome sequences of *C. ningqiangense* have been submitted to GenBank and the accession numbers are provided in Table 1.

Phylogenetic analyses were conducted using the Maximum Likelihood (ML) method. DNA sequence markers were aligned individually using MAFFT (Katoh and Standley 2013). The aligned sequences were then manually adjusted using trimAI (Capella-Gutiérrez et al. 2009). The concatenation of chloroplast CDS sequences and the construction of the phylogenetic tree were completed using PhyloSuite (Zhang et al. 2020). The ML phylogenetic tree was generated using IQ-TREE with ultrafast bootstrap support of 1000 replicates (Nguyen et al. 2015). The phylogenetic trees were edited and visually optimised using ITOL (Zhou et al. 2023).

## Results

### Morphological comparison

**Figure 1.**
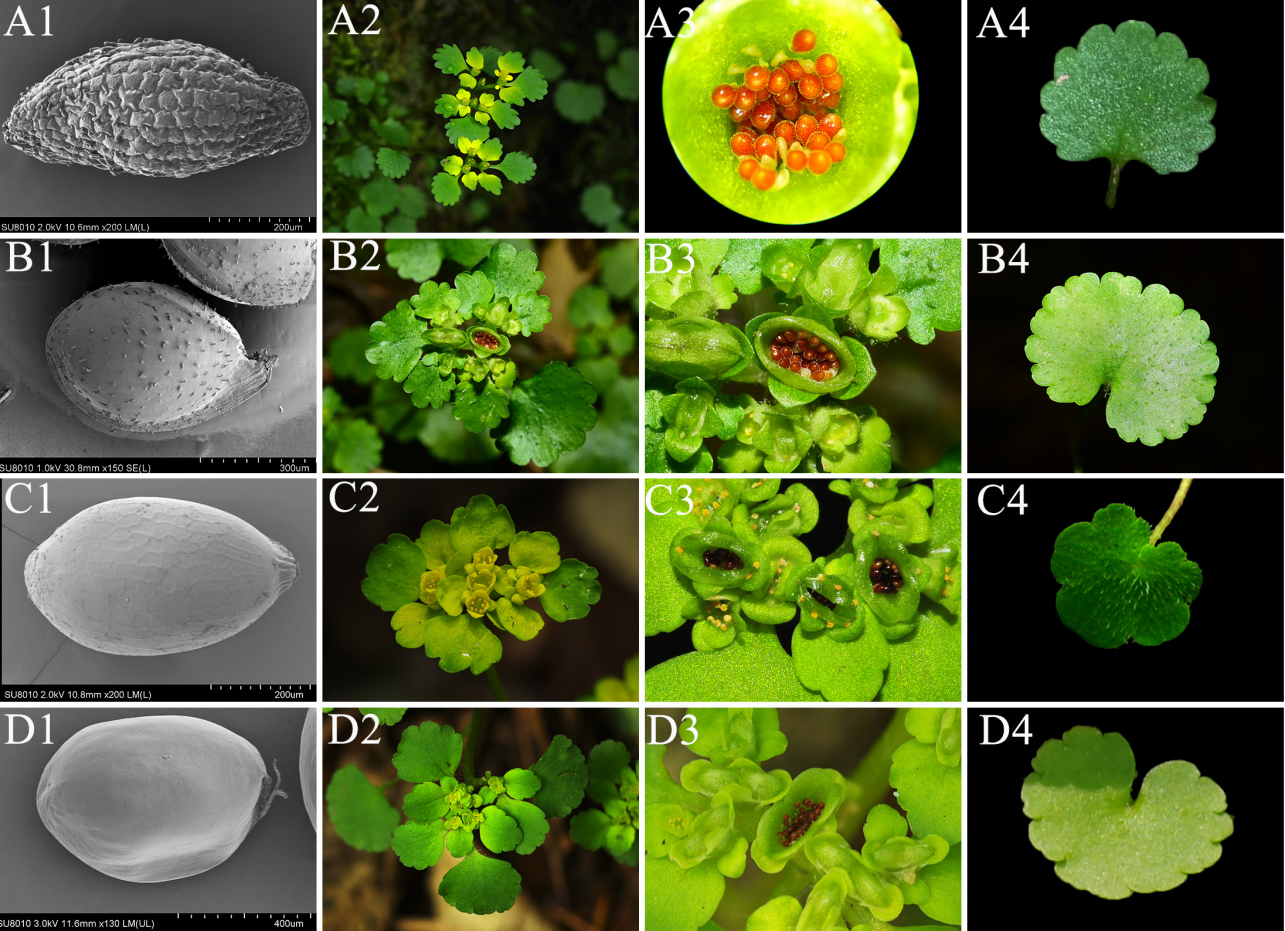
Mophological comparison of *C. ningqiangense* (**A1, A2, A3, A4**), *C. japonicum* (**B1, B2, B3, B4**), *C. serreanum* (**C1, C2, C3, C4**) and *C. alternifolium* (**D1, D2, D3, D4**). Seed surface (**A1, B1, C1, D1**); Inflorescence (**A2, B2, C2, D2**); Seed colour (**A3, B3, C3, D3**); Basal leaves (**A4, B4, C4, D4**).

*C. ningqiangense* is morphologically similar to *C. alternifolium*, *C. serreanum* and *C. japonicum*. However, the basal leaves and cauline leaves of *C. ningqiangense* are essentially consistent in mature plants. We can distinguish *C. ningqiangense* from other species by its leaf shape, yellow and loose inflorescences and a stem base that lacks any stolons, bulbils or bulbs. Seed colour and surface appendages are also important taxonomic characteristics for *Chrysosplenium*. We observed the seed colour and conducted a scanning electron microscope (SEM) analysis of the seeds, discovering that the reddish-brown seeds of *C. ningqiangense* have a tessellate and papillate surface. This characteristic differs significantly from those of *C. alternifolium*, *C. serreanum* and *C. japonicum*, thereby confirming that *C. ningqiangense* is a distinct new species. The key morphological feature comparisons are shown in Fig. 1 and Table 2.

**Table 2.** Mophological comparison of *C. ningqiangense*, *C. japonicum*, *C. serreanum* and *C. alternifolium.*

Characters	*C. ningqiangense*	*C. japonicum*	*C. serreanum*	*C. alternifolium*
**Stem base**	without stolons, bulbils, or bulbs	bulbils and bulbs	stolons	stolons
**Basal leaves**	petiole 1–2.5 cm, base truncate or broadly cuneate	petiole 1.5–8 cm, base deeply cordate or nephroid	petiole 2.5–4 cm, base nephroid	petiole 1.5–5 cm, base nephroid
**Cauline leaves**	abaxially subglabrous, adaxially pubescent	abaxially subglabrous, adaxially pilose	sparsely pilose	pubescent on both surfaces
**Inflorescence**	loose	compact	compact	compact
**Flower color**	yellow	green	yellow	greenish yellow
**Seed**	tessellate and papillate surface, reddish brown	papillate surface, reddish brown	tessellate surface, dark brown	smooth, chestnut brown

### Phylogenetic analysis

**Figure 2.**
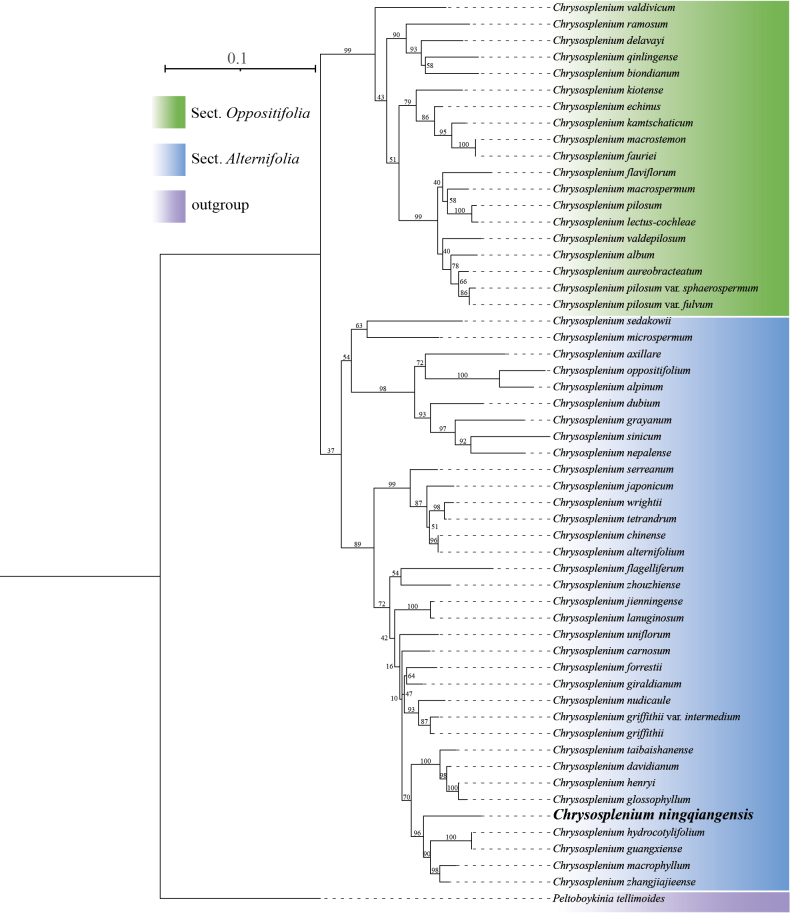
Phylogenetic placement of *C. ningqiangense* (bold representation) using the maximum likelihood (ML) method, based on nrITS. The bootstrap values are displayed above the branches.

**Figure 3.**
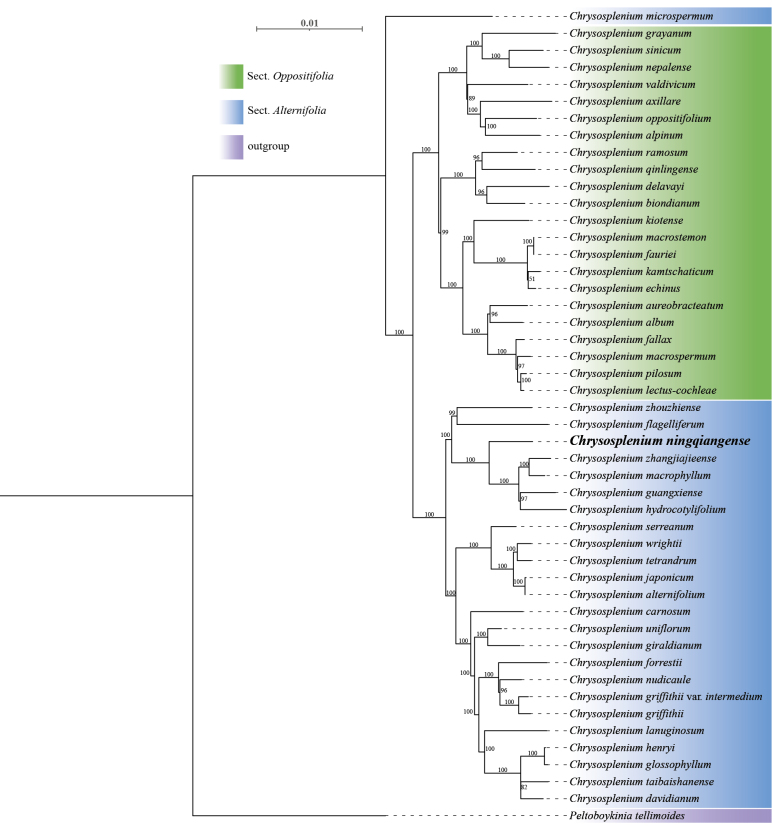
Phylogenetic placement of *C. ningqiangense* (bold representation) using the maximum likelihood (ML) method based on cpDNA. The bootstrap values are displayed above the branches.

The phylogenetic trees constructed, based on chloroplast CDS and nrITS, exhibit essentially consistent topologies (Figs 2, 3). Both analyses place *C. ningqiangense* in the same clade, although they show minor differences in supporting the monophyly of subgenus *Gamosplenium*. The phylogenetic relationships indicate that *C. ningqiangense* clusters with *C. hydrocotylifolium* H.Lév. & Vaniot, *C. guangxiense* H. G.Ye & G. C.Zhang, *C. macrophyllum* Oliv. and *C. zhangjiajieense* X. L.Yu, Hui Zhou & D. S.Zhou and does not form a sister group with any other species within *Chrysosplenium*. The result suggests that *C. ningqiangense* should also be treated as a new species at the molecular level. Although the phylogenetic relationships do not align perfectly with morphological inferences, all the species belong to the subgenus *Gamosplenium*, which effectively corroborates the taxonomic treatment of *C. ningqiangense* as a new species.

### Taxonomic treatment

#### Chrysosplenium ningqiangense

Taxon classificationPlantaeSaxifragalesSaxifragaceae

Hong Liu sp. nov.

B09C975D-78EA-567B-A538-AE40C5835136

urn:lsid:ipni.org:names:77378270-1

Fig. 4

##### Type.

China • Shannxi: Ningqiang County, Hanshuiyuan Forest Park, 32°45'2.628"N, 106°11'7.104"E, 1720 m elev, 14 April 2024, *Hong Liu LH2024041401* (holotype, HIB[HIB0259357] (Fig. 5); isotypes, HIB[HIB0259359], HIB[HIB0259358], HIB[HIB0259360], HIB[HIB0259361], HSN[HSN13551], HSN[HSN13552], HSN[HSN13553], HSN[HSN13554]).

**Figure 4.**
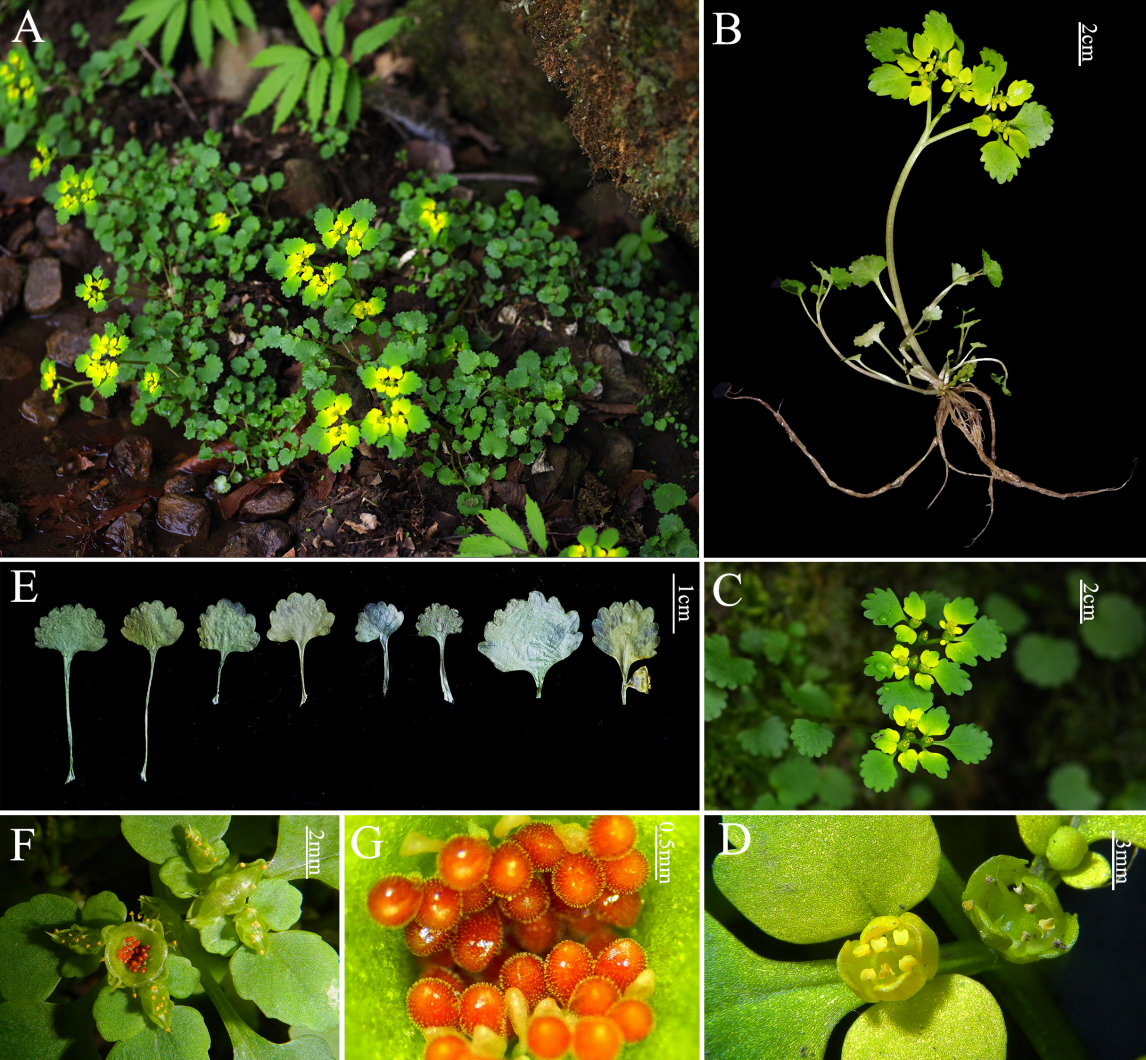
*Chrysosplenium ningqiangense*. **A**. habit; **B**. flowering whole plant; **C**. inflorescence; **D**. flowers; **E**. leaves (1–4 cauline leaves; 5–6 basal leaves; 7–8 bracteal leaves); **F**. capsule; **G**. seeds.

**Figure 5.**
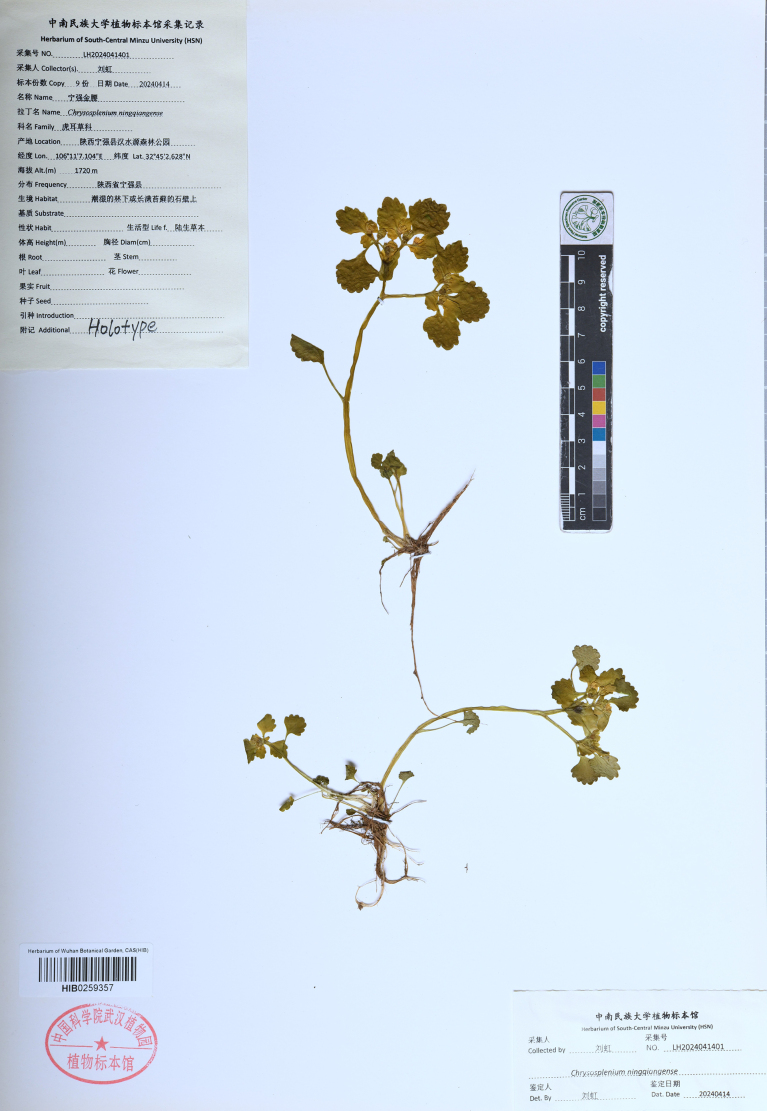
Photograph of the herbarium specimens of *C. ningqiangense*.

##### Diagnosis.

*C. ningqiangense* resembles *C. alternifolium*, *C. serreanum*, and *C. japonicum* but differs in leaf shape, yellow and loose inflorescences, and the absence of stolons, bulbils or bulbs at the stem base. Additionally, its reddish-brown seeds have a tessellate and papillate surface.

##### Description.

Perennial herbs. 10–18 cm tall, erect, hermaphroditic. Roots fibrous, diaphanous, without stolons, bulbils or bulbs. Flowering stems 1–2, erect, 10–15 cm long, tetragonal in the cross-section, with about 0.5 mm wide acies, glabrous, light green to green, sometimes sprinkled with red spots, with 1–4 sterile branches arising from the base; sterile branches 1–7 cm long, tender, glabrous. Leaves alternate, basal and cauline, simple, homomorphic, estipulate, petiolate. Basal leaves 3(2)-4, petiole 10–25 mm long, base truncate or broadly cuneate, middle and lower parts with sparse white tomentose, blade 5–12 × 7–15 mm, flabelliform or suborbicular. Cauline leaves of flowering stem(s) 1–4, alternate; petiole 10–25 mm long; blade 6–8 × 8–12 mm, flabelliform, abaxially subglabrous, adaxially pubescent, apex obtuse, base truncate or broad cuneate, margins 7–10 crenate, inconspicuous leaf veins. Leaves of sterile branches 3–4, alternate, gradually enlarging from the bottom up, mostly clustered at the top; petiole 5–15 mm long, subglabrous; blade 5–13 × 5–10 mm, flabelliform, flowering subglabrous, adaxially white bristles ca. 1 mm in summer, abaxially glabrous; apex obtuse, base truncate, margins 8–13 crenate, inconspicuous leaf veins. Inflorescence with 4–14 flowers, a loose cyme, surrounded by leaf-like bracts; top view 5–8 cm wide, glabrous. Bracteal leaves bright yellow to greenish-yellow; broadly ovate, obovate to orbicular, glabrous, base broadly cuneate to oblique broadly cuneate, triangularly arranged, one in the middle distinctly larger than the two sides; often in 2–3 whorls, the petiole of the first whorl of bracts 5–8 mm long, blade 5–12 × 4–12 mm, margins crenate(7–11 teeth); the petiole of the second whorl of bracts 2–5 mm long, blade 3–5 × 2–4 mm, margins serrulate (3–5 teeth). Flowers tetramerous, actinomorphic, diameter 2–3 mm; sepals 4 (2 pairs), erect and yellow during flowering, green during fruiting, 2.6–3.9 ×1.8–2.2 mm, ovate, glabrous, apex obtuse to acute; petals absent; stamens 8, biseriate, ca. 2–3 mm long, shorter than sepals; filaments tenuous, erect, ca. 2 mm long; anthers yellow, 2-locular, longitudinally dehiscent; ovary superior, 2-locular, zygomorphic; stigmas 2, equal, ca. 2 mm long, as long as filaments. Capsule 2-lobed, bright green, 5–7 mm × 3–4 mm, glabrous, apex truncate and retuse, both segments equal in size, dehiscing apically at maturity, rostrum ca. 1–2 mm long, with 30–40 seeds per capsule; Seeds reddish-brown, sub-ovoid, with a raised median suture on one side, ca. 550–640 × 350–450 µm and tessellate and papillate surfaces.

##### Distribution and habitat.

*C. ningqiangense* is endemic to Hanshuiyuan Forest Park, Ningqiang County, Shaanxi Province, with three populations totalling approximately 300–400 plants. It grows on wet, moss-rich stone walls or ground.

##### Preliminary conservation assessment.

*C. ningqiangense* is preliminarily classified as Endangered (EN B1b(v); C2a(i)) due to its limited population size and restricted distribution according to the guidelines of the International Union for Conservation of Nature (IUCN) Red List Categories and Criteria (IUCN 2022).

##### Etymology.

The specific epithet refers to the type locality, Ningqiang County.

##### Vernacular name.

“宁强金腰” (Ningqiang Jinyao).

##### Phenology.

Flowering from mid-March to mid-April; fruiting from mid-April to mid-May.

## Discussion

Morphological and phylogenetic analyses support the classification of *C. ningqiangense* as a new species within Subgen. *Gamosplenium* (Pan and Ohba 2001). The species can be distinguished from others by its leaf shape, yellow and loose inflorescences and the absence of stolons, bulbils or bulbs at the stem base. Additionally, *C. ningqiangense* has reddish-brown seeds with a tessellate and papillate surface. Furthermore, *C. ningqiangense* has a relatively early blooming period (March to May) compared to other species in *Chrysosplenium*.

Although clustering with *C. hydrocotylifolium*, *C. guangxiense*, *C. macrophyllum* and *C. zhangjiajieense* in the phylogenetic tree, *C. ningqiangense* exhibits significant morphological differences, particularly in leaf size and shape. This discrepancy may reflect the need for further taxonomic refinement within *Chrysosplenium*. After a brief examination of the morphological features of *C. hydrocotylifolium*, *C. guangxiense*, *C. macrophyllum* and *C. zhangjiajieense*, we found that although these species differ significantly in traits, such as plant size and leaf morphology, they exhibit consistent characteristics in finer structures, particularly the stem base and seed surface. For instance, all these species lack stolons, bulbils or bulbs at the stem base and their seeds all possess a papillose surface. In taxonomy, qualitative characters are often more stable than quantitative ones. We therefore speculate that these features may serve as key diagnostic characters for the classification of *Chrysosplenium*, a conclusion that is consistent with Pan’s taxonomic treatment (Pan and Ohba 2001; Liu et al. 2016; Fu et al. 2024). In this study, phylogenetic trees were constructed separately using nrITS and cpDNA. The nrITS-based tree shows that Subgen. *Gamosplenium* forms a monophyletic group, but with low support (37%). The cpDNA-based tree shows a non-monophyletic status of Subgen. *Gamosplenium*, which aligns with Fu et al. (2024). This implies that the classification of *Chrysosplenium* needs further revision.

The discovery of *C. ningqiangense* enriches the diversity of *Chrysosplenium* and provides new insights into the genus’ phylogenetic relationships. Recent discoveries of new *Chrysosplenium* species in China (Kim et al. 2019; Fu et al. 2020, 2021, 2024) highlight the need for continued exploration of the genusin the region.

## Supplementary Material

XML Treatment for Chrysosplenium ningqiangense

## Additional information

### Use of AI

No use of AI was reported.

### Author ORCIDs

Cai-Quan Shen https://orcid.org/0009-0009-9091-4650

Ke-Hui Zhang https://orcid.org/0009-0004-6165-7126

Tian-Ge Yang https://orcid.org/0000-0003-1681-1767

Hong Liu https://orcid.org/0000-0001-7227-2476

### Data availability

All of the data that support the findings of this study are available in the main text or Supplementary Information.

## Supplementary materials

Supplementary material 1 Specimen information for all samples used in the phylogenetic reconstruction Data type xlsx https://binary.pensoft.net/file/1578517This dataset is made available under the Open Database License (http://opendatacommons.org/licenses/odbl/1.0/). The Open Database License (ODbL) is a license agreement intended to allow users to freely share, modify, and use this Dataset while maintaining this same freedom for others, provided that the original source and author(s) are credited. Cai-Quan Shen, Yong Wang, Ke-Hui Zhang, Tian-Ge Yang, Rui Qin, Hong Liu
